# Supplementary material for: Downregulating Mitochondrial DNA Polymerase γ in the Muscle Stimulated Autophagy, Apoptosis, and Muscle Aging-Related Phenotypes in Drosophila Adults
Source: Biomolecules. 2022 Aug 11;12(8):1105. doi: 10.3390/biom12081105 (PMC9405705; doi:10.3390/biom12081105)
Supplement: Supplementary file 1 [file biomolecules-12-01105-s001.zip › biomolecules-1832970-supplementary.pdf]

Supplementary figure legends

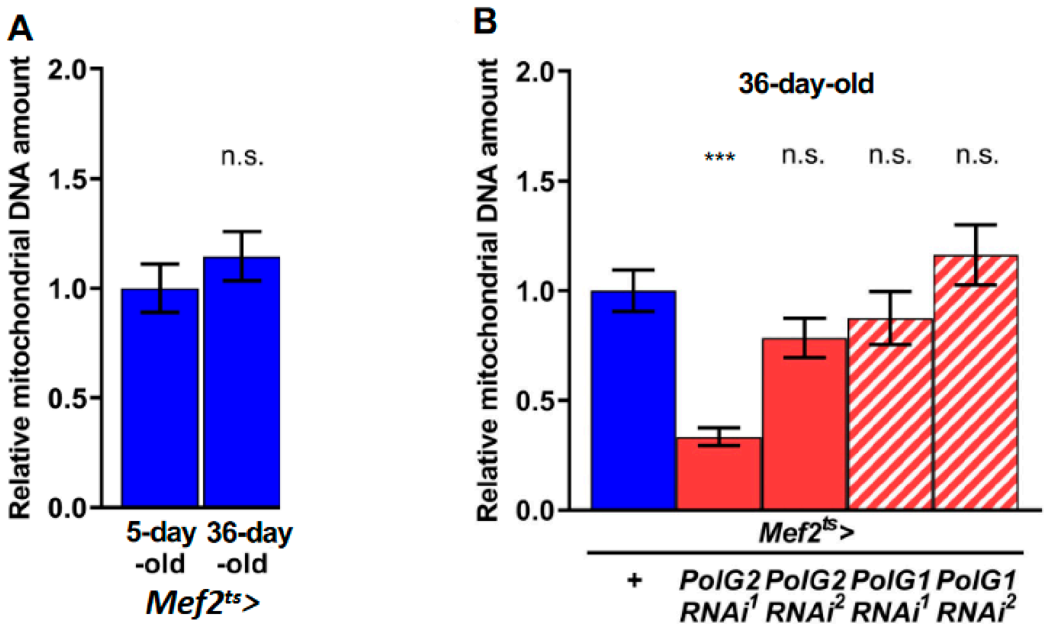

Fig. S1. Quantification of mitochondrial DNA relative to nuclear DNA in adult thoraxes harboring *PolG1RNAi* and *PolG2RNAi*

(A) Quantification of the average amount of mitochondrial DNA (mtDNA) relative to nuclear DNA in the thoraxes of 5-day-old and 36-day-old adults (*Mef2^{ts}*>+) by qPCR. The mtDNA amount in 5-day-old control adults is presented as 1.0. (B) Relative amount of mtDNA relative to nuclear DNA in the thoraxes from 36-day-old control adults (*Mef2^{ts}*>+), and adults harboring *PolG2RNAi* (*Mef2^{ts}>PolG2RNAi^{1}* and *Mef2^{ts}>PolG2RNAi^{2}*) and *PolG1RNAi* (*Mef2^{ts}>PolG1RNAi^{1}* and *Mef2^{ts}>PolG1RNAi^{2}*) by qPCR. The mtDNA amount in control adults at 36-day-old is presented as 1.0. (A; n=3 (triplicates), B; n=9 (three biological triplicates). Welch's *t*-test and one-way ANOVA with Bonferroni's multiple comparisons test were applied to compare the mean differences in A and B, respectively. ***$p < 0.001$, ns; not significant $p > 0.05$.

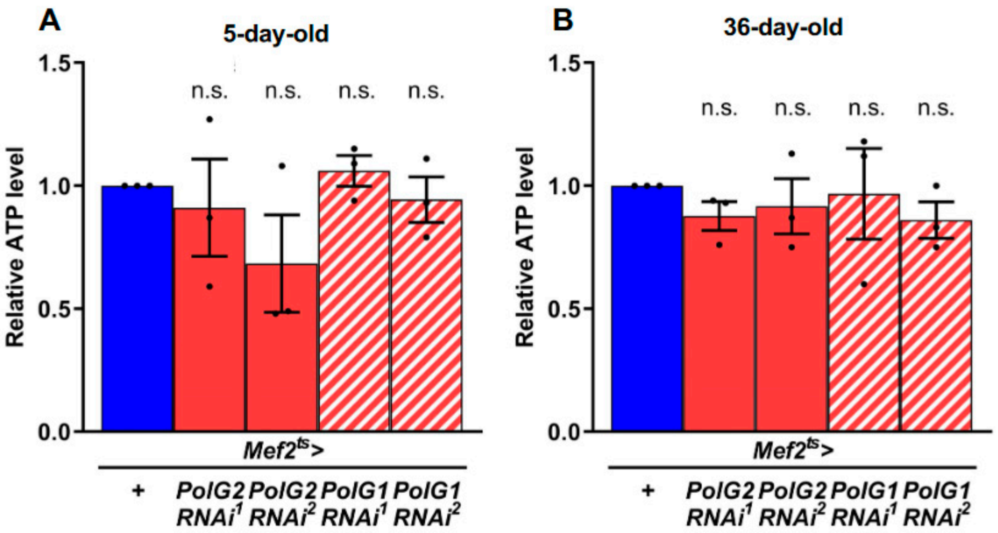

Fig. S2. Relative levels of ATP contained in adult thoraxes harboring *PolγRNAi*

(A, B) Quantification of ATP levels in 10 thoraxes using a luciferase assay. Thoraxes of 5-day-old (A), and 36-day-old (B) control adults, and adults harboring muscle specific *PolG2RNAi* (*Mef2^{ts}>PolG2RNAi^{1}*, *Mef2^{ts}>PolG2RNAi^{2}*) and *PolG1RNAi* (*Mef2^{ts}>PolG1RNAi^{1}* and *Mef2^{ts}>PolG1RNAi^{2}*) were prepared. The average ATP content was normalized to total protein content. (n=3 (three repeated assays), n.s.; not significant, one-way ANOVA with Bonferroni's multiple comparisons test). Error bar; s.e.m.

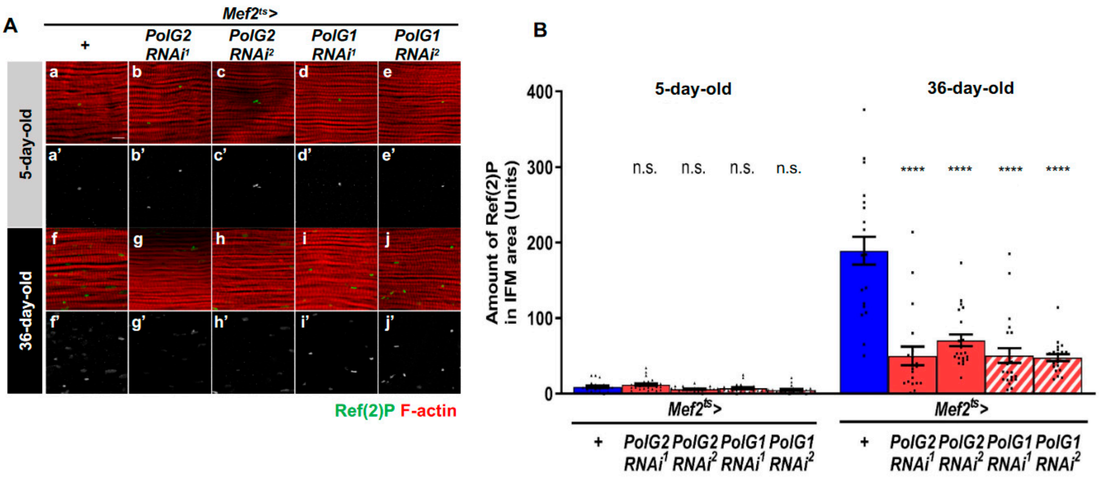

Fig. S3. Immunostaining and quantification of autophagy adaptor protein, Ref(2)P in adult IFMs harboring the muscle-specific *PolγRNAi*

(A) Immunostaining of IFMs from 5 and 36-day-old adults (A) or 5- and 36-day-old control adults and adults harboring muscle-specific *PolG2RNAi* and *PolG1RNAi* (*Mef2^{ts}>PolG2RNAi^{1}*, *Mef2^{ts}>PolG2RNAi^{2}*,

Mef2^{ts}>*PolG1RNAi*^{1} and *Mef2*^{ts}>*PolG1RNAi*^{2}) with anti-Ref(2)P antibody (green) and phalloidin for F-actin (red). Scale bar: 10 μm. (B) Quantification of the Ref(2)P foci in the IFMs of the 5- and 36-day-old control flies (*Mef2*^{ts}>+, (n=21 for 5-day-old, n=20 for 36-day-old) and flies harboring muscle-specific *PolG2RNAi* and *PolG1RNAi* (*Mef2*^{ts}>*PolG2RNAi*^{1} (n=21 for both age groups), *Mef2*^{ts}>*PolG2RNAi*^{2} (n=22, 20), *Mef2*^{ts}>*PolG1RNAi*^{1} (n=22, 21) and *Mef2*^{ts}>*PolG1RNAi*^{2} (n=24, 22). Total pixels of the foci per single confocal optical fields were quantified. The number is represented using 10 pixels as a unit. (****$p<0.0001$, one-way ANOVA with Bonferroni's multiple comparisons test). Error bars; s.e.m.

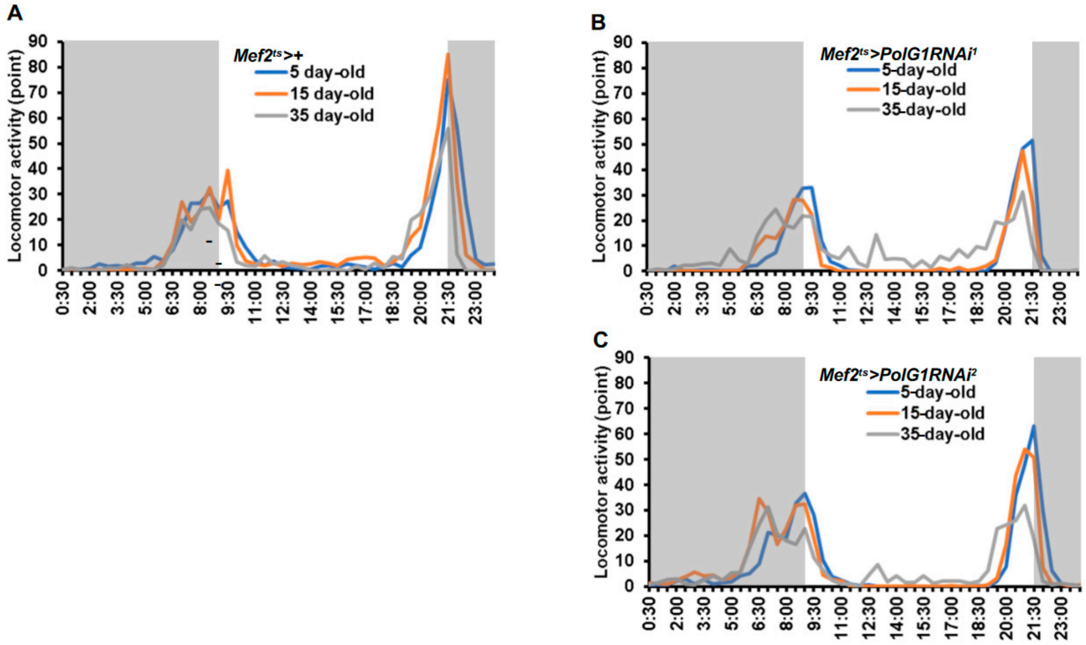

Fig. S4. Assay of daily locomotor activity of adults harboring muscle-specific *PolγRNAi*

(A-C) Daily locomotor activity of 5-, 15-, and 35-day-old flies under a 12-h/12-h light-dark cycle. (A) Control flies (*Mef2*^{ts}>+), (B, C) flies harboring muscle-specific *PolG1RNAi* (*Mef2*^{ts}>*PolG1RNAi*^{1} (B), *Mef2*^{ts}>*PolG1RNAi*^{2} (C). Higher and more pointed peaks of locomotor activity are seen at the end of the daytime, just before light was turned off (around 21:00) and another lower and relatively broader peak is seen around 08:00, just before the lighting was switched on. *PolG1RNAi* flies exhibited a remarkable decline in locomotor activity at a higher peak with age compared with controls. The 35-day-old flies harboring *PolG1RNAi* (*Mef2*^{ts}>*PolG1RNAi*^{1} and *Mef2*^{ts}>*PolG1RNAi*^{2}) showed relatively higher locomotor activity during the daytime (B, C), while control adults at the same age showed the background activity level (A) (n=20 flies in every genotype).
